# Cross-polarized and stable second harmonic generation from monocrystalline copper

**DOI:** 10.1515/nanoph-2025-0388

**Published:** 2025-11-19

**Authors:** Elif Nur Dayi, Omer Can Karaman, Diotime Pellet, Alan R. Bowman, Giulia Tagliabue

**Affiliations:** Laboratory of Nanoscience for Energy Technologies (LNET), STI, École Polytechnique Fédérale de Lausanne, 1015 Lausanne, Switzerland; Department of Physics, University of Oxford, Oxford OX1 3PU, UK

**Keywords:** nonlinear spectroscopy, monocrystalline microflakes, second harmonic generation (SHG), copper, stability, anisotropy

## Abstract

Second-harmonic generation (SHG) is a powerful surface-specific probe for centrosymmetric materials, with broad relevance to energy and biological interfaces. Plasmonic nanomaterials have been extensively utilized to amplify this nonlinear response. Yet, material instability has constrained most studies to gold, despite the significance of plasmonic metals such as copper for catalysis. Here, we demonstrate stable and anisotropic SHG from monocrystalline copper, overcoming long-standing challenges associated with surface degradation. By leveraging an on-substrate synthesis approach that yields atomically flat and oxidation-resistant Cu microflakes, we enable reliable SHG measurements and reveal a strong cross-polarized response with *C*_3*v*_ surface symmetry. The SHG signal remains stable over 3 h of continuous femtosecond excitation, highlighting the remarkable optical robustness of the Cu microflakes. These results reinforce the viability of monocrystalline Cu as a robust platform for nonlinear nanophotonics and surface-sensitive spectroscopy, expanding the range of copper-based optical applications.

## 1 Introduction

Nonlinear optical characterization has emerged as a powerful approach to probe the symmetry and structural properties of nanomaterials and to control light–matter interactions at the nanoscale [1], [2]. Among nonlinear processes, second harmonic generation (SHG) is particularly valuable due to its sensitivity to inversion symmetry breaking and consequently its surface-selective nature in centrosymmetric materials [3]. As a non-destructive technique with broad applicability, SHG is widely employed in plasmonics [2], [4], nanophotonics [5], [6], biosensing [7], [8], and heterogeneous catalysis [9], [10], [11].

SHG has been extensively studied in materials including noble metals such as gold [12], [13], silver [4], [14], [15], as well as in two-dimensional materials [16], [17] and transition metal dichalcogenides [6], [18], [19] in the past decades. However, the nonlinear optical behavior of copper, a material of paramount importance for energy conversion [20], catalysis [21], [22] and sensing [23], [24], remains largely unexplored. This is mainly due to its metastable surface chemistry and its tendency to undergo oxidation under ambient conditions within a few hours [25], which can significantly influence the measurements [26], [27], [28], [29]. Moreover, previous investigations have focused almost exclusively on polycrystalline Cu films [15], [26], [28], [30], [31], where randomly oriented grains average out any anisotropic response and limit insights into the fundamental nature of Cu based SHG behavior.

Direct access to the intrinsic nonlinear optical response of monocrystalline Cu offers a unique opportunity to investigate SHG in centrosymmetric metals beyond gold, thereby expanding the fundamental understanding of second-order processes in plasmonic systems. In this work, leveraging our recently reported oxidation-resistant Cu monocrystalline microflakes [32], we report reliable SHG measurements from monocrystalline Cu(111) surfaces under ambient conditions. Using power- and polarization-dependent measurements, we demonstrate that Cu microflakes exhibit dominant co- and cross-polarized SHG components, with the cross-polarized signal maximized when the incident polarization is aligned with the crystal axis and the co-polarized signal maximized when the polarization is perpendicular to a crystal axis. The observed threefold polarization dependence is consistent with *C*_3*v*_ symmetry, as expected for fcc (111) surfaces [29]. In contrast, polycrystalline Cu films display significantly weaker, isotropic emission. These findings highlight the viability of oxidation-resistant monocrystalline Cu microflakes as a robust platform for nonlinear nanophotonics and surface-sensitive spectroscopy, expanding the design space for copper-based optical and catalytic materials.

## 2 Methods

### 2.1 Sample fabrication

Cu microflakes were grown using the optimized protocol described in our previous work [32]. Polycrystalline Cu films were sputter-coated onto glass substrates at room temperature using an Alliance-Concept DP650 system with an average deposition rate of 1.2 Å s^−1^. For each measurement, a fresh polycrystalline Cu film was prepared. To minimize risk of oxidation, the sample was immediately transferred from the deposition chamber into a nitrogen-filled container for transporting and storage. It was exposed to air only at the time of SHG measurement. Flake samples were also stored in a nitrogen-purged environment in between measurements.

### 2.2 Second-harmonic generation (SHG) measurements

SHG measurements were performed using a customized NT&C setup consisting of an inverted microscope (Nikon Eclipse T2) coupled to a spectrometer (Princeton Instruments HRS-500). A 1,030 nm fs laser (NKT Origami) was focused onto the sample with a 60× long working distance objective (Thorlabs, NA = 0.7), and the incident power was adjusted using a variable neutral density filter (Thorlabs). The emitted SHG signal was first directed by a 750 nm dichroic mirror (Edmund Optics), which reflects the SHG light (at 515 nm) and transmits the residual fundamental (1030 nm). An 850 nm short-pass filter placed after the dichroic further suppressed any remaining fundamental laser. The filtered SHG signal was then collected through a 4-f lens system before reaching the spectrometer or CCD camera for detection. Measured SHG intensities were normalized by excitation power, illumination area, and integration time to allow comparison between samples.

Power-dependent measurements were acquired by varying the fundamental laser intensity from 0.35 to 2.15 mW/μm^2^, and the SHG intensity was fitted on a log–log scale to extract the power-law exponent.

For polarization-resolved SHG measurements, the sample was rotated in 5° steps using a motorized stage, with 30 s of exposure at each condition. The coordinate system was defined with the *x*- and *y*-axes lying in the sample plane and the *z*-axis normal to the surface, while the polarization angle was defined as the in-plane polarization angle (*φ*) for both emission and excitation. Co- and cross-polarized emission components were collected using a polarizer placed before the spectrometer.

## 3 Results & Discussion

Monocrystalline Cu microflakes grown using our recently developed on-substrate synthesis method [32] reach lateral dimensions of tens of micrometers. Advanced structural characterization has shown that they possess atomically flat top surfaces with a well-defined (111) surface orientation. Remarkably, these flakes also exhibit long-term resistance to surface oxidation: no oxide formation is observed even after one month, enabled by the presence of an atomically thin bromide adlayer.

**Figure 1:**
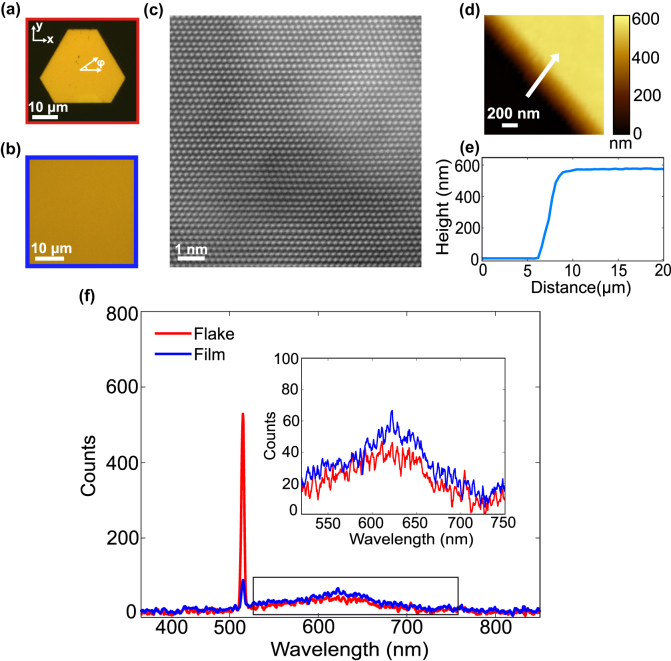
Structural and optical characterization of monocrystalline and polycrystalline copper samples. Bright-field images of (a) a monocrystalline Cu microflake grown on a glass substrate and (b) a sputtered polycrystalline Cu thin film. (c) High-angle annular dark-field (HAADF) transmission electron microscopy (TEM) image of the Cu microflake, confirming its single-crystalline structure. (d) Atomic force microscopy (AFM) scan of the microflake edge; the white arrow indicates the line used to extract the height profile in (e), revealing a step height of ∼580 nm. (f) SHG emission spectra collected under 1030 nm excitation at 2 mW/μm^2^ for monocrystalline microflakes (red) and polycrystalline films (blue) of similar thickness. Inset shows the expanded view of the two-photon photoluminescence background from 550 to 750 nm range.

Figure 1a shows a bright-field image of a representative Cu microflake, with its single-crystalline nature evident from the truncated triangular shape. Polycrystalline Cu films with varying thicknesses were also prepared to enable direct comparison of SHG signals between monocrystalline and polycrystalline samples (Figure 1b). The high-angle annular dark-field scanning transmission electron microscopy (HAADF-STEM) image in Figure 1c further confirms the single-crystalline face-centered cubic structure of the flake.

A Cu(111) microflake with a measured thickness of 580 nm (Figure 1d), determined from the AFM height profile in Figure 1e, was compared to a polycrystalline film of comparable thickness (∼500 nm, estimated from deposition rate). Under 1,030 nm fs laser excitation, the resulting 515 nm SHG signal is significantly stronger in the monocrystalline microflake than in the polycrystalline film (Figure 1f). In addition, the broad spectral features observed between 550 nm and 650 nm in the flake spectrum (inset) suggest weak two-photon luminescence (TPL), consistent with interband and intraband transitions in Cu [33], [34], [35]. Stronger TPL signal is expected from the rougher surface of thin films due to enhanced local field confinement and increased surface area [34]. However, in some measurements (see Figure S3), the flake exhibited higher TPL intensity than the film, which may be attributed to variations in sample thickness.

**Figure 2:**
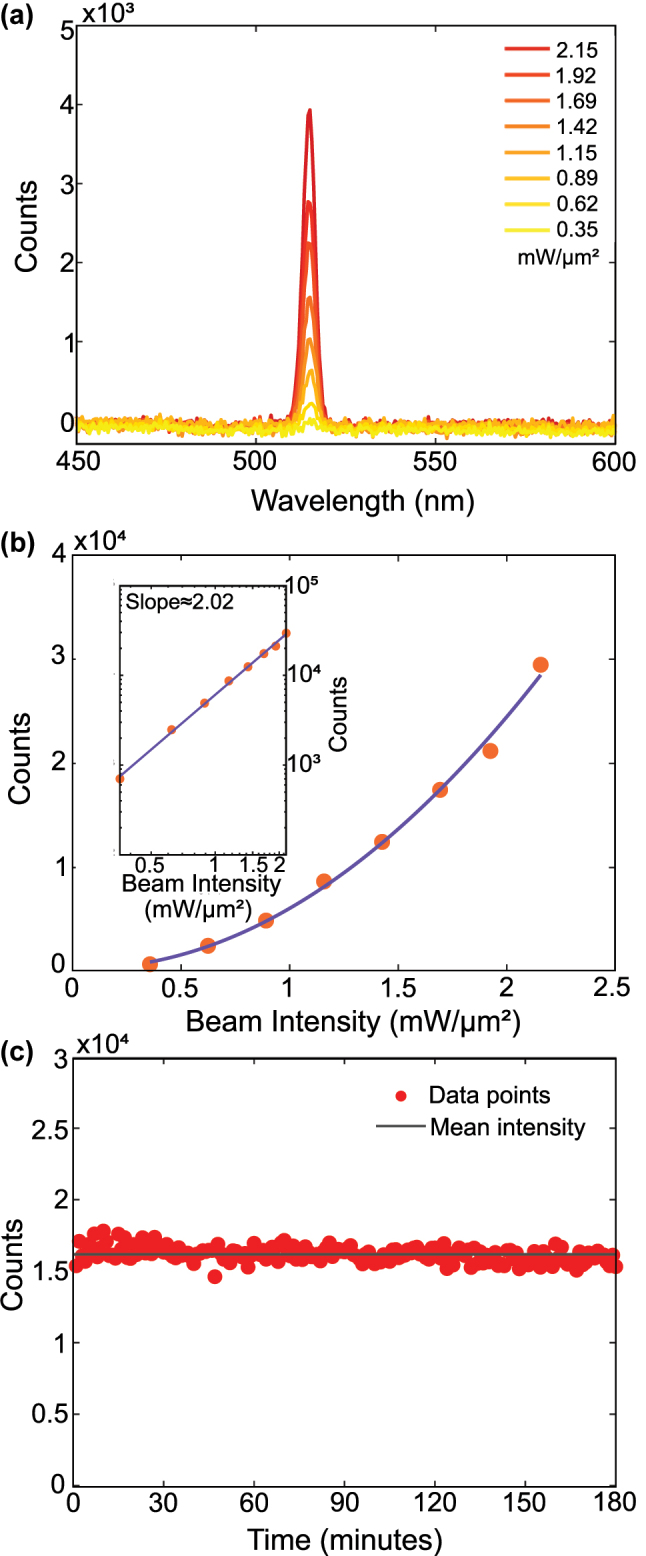
Power dependence and stability of second-harmonic generation from a Cu microflake. (a) SHG intensity from the Cu microflake under varying beam intensities of 0.35–2.15 mW/μm^2^. (b) Quadratic increase of SHG intensity with respect to the powers shown in part a. The inset shows the logarithm curve with a slope of 2.02 ± 0.08, which strongly agrees with the theoretical value of 2 expected for second-order processes. (c) SHG stability measurement of a Cu microflake under ambient conditions. The sample was continuously illuminated at 2 mW/μm^2^, and one spectrum was recorded every minute over a 3-h period. Red dots represent the integrated SHG intensity between 500 and 525 nm for each spectrum, while the black line indicates the mean intensity (∼16,000 counts). The signal remained stable within ±3.2 % throughout the measurement, demonstrating excellent photostability of the flake under prolonged laser exposure.

To confirm the second-order nature of the SHG and ensure the microflake stability under illumination, we performed power-dependent and time-dependent SHG measurements. Figure 2a and b show the increase in SHG intensity with increasing laser intensity from 0.35 to 2.15 mW/μm^2^. The log–log plot in the inset of Figure 2b yields a slope of ≈2.02 ± 0.08, in excellent agreement with the expected value of 2 for a second-order process.

The stability of the Cu microflakes under continuous illumination was evaluated by monitoring the SHG intensity over a 3 h period at a beam intensity of 2 mW/μm^2^, as shown in Figure 2c. Bright-field images of the Cu microflake before and after illumination are included in Figure S4, showing no visible change to the surface. Moreover, given the surface-sensitive nature of SHG, any photo-induced oxidation or structural damage would be expected to result in a measurable change in signal intensity. The recorded SHG signal remained stable throughout the entire duration, exhibiting only ±3.2 % fluctuation relative to the mean intensity (≈16,000 counts). This confirms the remarkable optical stability of the microflakes and demonstrates that stable SHG can be obtained from monocrystalline copper.

**Figure 3:**
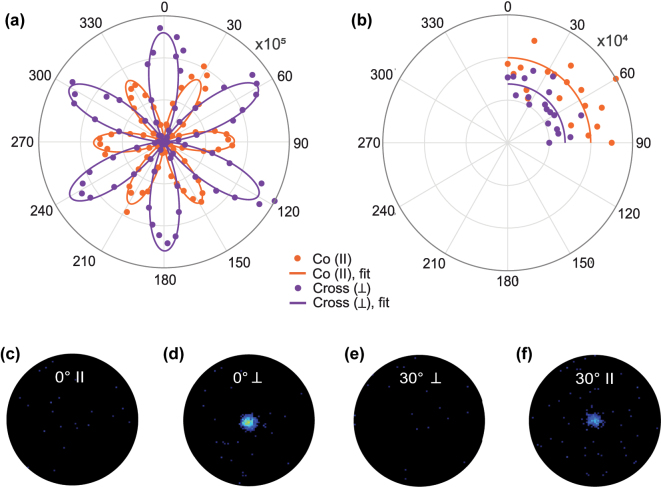
Symmetry-driven polarization behavior of SHG from monocrystalline Cu microflakes. (a) Polarization-dependent intensity of the second harmonic for the monocrystalline Cu microflake at 2.5 mW/μm^2^ excitation. Cross-polarized emission (purple), i.e. signals emitted perpendicular to the fundamental polarization, and co-polarized emission (orange), which is parallel to the fundamental polarization are shown. The data were fitted using the model by Sipe et al. [36]. (b) Polarization-dependent intensity measured for the polycrystalline Cu film measured using the same experimental configuration. The response of the polycrystalline film is represented by the average of all data points. (c–f) Fourier images of the SHG recorded in co-polarized and cross-polarized detection for crystal orientations *φ* = 0° and *φ* = 30°, using a 60× objective (NA = 0.7): (c) *φ* = 0°, co-polarized; (d) *φ* = 0°, cross-polarized; (e) *φ* = 30°, cross-polarized; (f) *φ* = 30°, co-polarized. No significant SHG signal was detected in panels (c) and (e). The bright central feature in (d) confirms dominant SHG emission in the cross-polarized channel at *φ* = 0°, attributed to strong in-plane anisotropic nonlinear polarization components. When the crystal orientation is changed to *φ* = 30°, the dominant SHG signal appears in the co-polarized channel (f). In both cases, the SHG emission is concentrated near the center of the Fourier plane, indicating that the emission is predominantly directed normal to the surface of the flake.

To understand the fundamental nature of the SHG signal, we performed polarization-dependent measurements on monocrystalline Cu microflakes and their polycrystalline Cu counterparts, for which the bright-field micrographs were shown in Figure 1a and b. In these measurements, we fixed the incident beam polarization and rotated the sample while recording the cross and co-polarized SHG intensities. Further details of these measurements are included in the Methods 2 section and the resulting polar plots are shown in Figure 3.

The six-petal SHG pattern in Figure 3a confirms the anisotropic surface response of the Cu microflake and indicates that its surface symmetry corresponds to the point group *C*_3*v*_ [29], [37], characterized by threefold rotational symmetry. For a (111) surface with *C*_3*v*_ symmetry, the inversion symmetry of the bulk is broken only at the surface, giving rise to a restricted set of non-vanishing second-order susceptibility tensor elements, specifically $\chi_{\mathrm{xxz}}^{\left(2\right)}=\chi_{\mathrm{yyz}}^{\left(2\right)}$ , $\chi_{\mathrm{zxx}}^{\left(2\right)}=\chi_{\mathrm{zyy}}^{\left(2\right)}$ , and $\chi_{\mathrm{zzz}}^{\left(2\right)}$ [38], [39], [40]. The interaction between these in-plane and out-of-plane tensor components leads to angular modulations of the SHG intensity that follow sin(3*φ*) and cos(3*φ*) dependencies, producing the characteristic six-petal pattern observed in the polar plots. This anisotropic response directly reflects the threefold rotational symmetry of the Cu(111) surface and has been extensively described for noble metal surfaces in both theoretical and experimental works [29], [31]. The observed *C*_3*v*_ pattern is consistent with observations reported for Au(111) flakes, where a similar SHG angular dependence was attributed to symmetry-allowed anisotropic tensor contributions on fcc (111) metal surfaces [41]. The SHG signal exhibits clear angular modulation in both co- and cross-polarized detection channels as the excitation polarization is rotated relative to the crystal axis. Different orientations probe distinct combinations of nonlinear susceptibility tensor elements; for instance, the co-polarized signal peaks near 30°, where the excitation field couples most strongly onto tensor elements such as $\chi_{\mathrm{xxx}}^{\left(2\right)}$ , corresponding to the dominant sin(3*φ*) terms in the fit and reflecting in-plane electric field coupling at the surface [31]. Minor amplitude differences at angles such as 30° and 330° likely stem from small alignment or focusing variations during rotation.

In the case of the polycrystalline Cu film, shown in the polar plot of Figure 3b, the absence of angular dependence is consistent with expectations, resulting from the averaging of randomly oriented grains under excitation by a beam with a spot size of approximately 2 μm [26].

We note that while the surface of the Cu(111) microflakes is terminated by an atomically thin bromide adlayer [32], its conformal and sub-nanometer nature allows for a nonlinear optical response that primarily reflects the underlying copper surface. This interpretation is reinforced by prior work showing that ultrathin CuBr flakes exhibit polarization-resolved SHG signatures that differ markedly from those observed here [42]. Notably, SHG spectroscopy is well established as highly sensitive to surface adsorption and even monolayer coverage of small molecules or ions can strongly modulate the SHG response [11], [40], [43]. The fact that our SHG signal remains stable over extended measurements (Figure 2c) and retains the expected anisotropic pattern (Figure 3a) indicates that the Cu(111) surface remains clean and chemically inert under our experimental conditions. We attribute this stability to the intrinsic bromide termination formed during growth, which passivates the surface without introducing perturbations detectable by SHG, unlike the effects typically induced by adventitious adsorbates or surface oxidation.

Further insight on the SHG signal from Cu monocrystalline flake is provided by the Fourier plane images shown in Figure 3c–f, which reveal the angular distribution of the SHG emission. These measurements were performed on a different Cu microflake and the corresponding polar plot is included in Figure S2, in good agreement with the polar plots in 3. The bright central spot observed in the cross-polarized channel at 0° (panel d), and similarly in the co-polarized channel at 30° (panel e), confirms that the SHG emission is directed predominantly along the surface normal (out-of-plane), while no significant SHG signal was detected in panels (c) and (f).

This symmetry-driven directionality could be exploited in future nonlinear optical devices that require polarization control or angular filtering of SHG signals [4], [44]. As established in the nonlinear optics literature [45], [46], SHG can be further enhanced in multilayer or hybrid systems through interference and field confinement effects. Therefore, the unique Cu (111) platform demonstrated here could be integrated within such multilayer architectures to achieve additional enhancement. Likewise, previous studies have shown that nanostructured and plasmonic Cu architectures can significantly enhance SHG through local field confinement and increased surface area [47], [48], [49], [50]. This work lays foundation towards integration of Cu(111) microflakes with such plasmonic architectures to couple intrinsic symmetry-driven SHG with extrinsic and resonant field enhancement.

## 4 Conclusions

In summary, we present a comprehensive study of the anisotropic nonlinear response of monocrystalline Cu with (111) surface orientation at ambient conditions, thanks to the distinct stability of the Cu microflakes under illumination. We confirm that the SHG measurements can determine the crystal structure, which for monocrystalline Cu shows point group *C*_3*v*_ characterized by threefold rotational symmetry, unlike a polycrystalline film. Furthermore, we observe a pronounced cross-polarized SHG emission from Cu, bridging the gap between the nonlinear optical behavior of Cu and Au microflakes as revealed by polarization-dependent measurements. Although recent studies on nonlinear metasurfaces have largely relied on gold for its stability and fabrication maturity [44], [45], our work reveals that copper can offer a viable alternative. With our demonstration of robust SHG from high-quality Cu microflakes, we aim to provide groundwork for earth-abundant and cost-effective copper-based nonlinear platforms. Overall, we envision these findings will promote new applications and research directions in the development of copper-based nonlinear plasmonic and nanophotonic devices, as well as foster better integration of SHG spectroscopy as a rapid characterization tool in energy conversion and photocatalysis [28], [51], [52], [53].

## Supplementary Material

Supplementary Material Details
