# Supplementary material for: Cross-polarized and stable second harmonic generation from monocrystalline copper
Source: Nanophotonics. 2025 Nov 19;14(25):4575–82. doi: 10.1515/nanoph-2025-0388 (PMC12714057; doi:10.1515/nanoph-2025-0388)
Supplement: Supplementary file 1 — Supplementary Material Details [file j_nanoph-2025-0388_suppl_001.pdf]

Supporting Information

Cross-polarized and Stable Second Harmonic Generation from Monocrystalline Copper

Elif Nur Dayi[1], Omer Can Karaman[1], Diotime Pellet[1], Alan R. Bowman[*2], and Giulia Tagliabue[†1]

[1]Laboratory of Nanoscience for Energy Technologies (LNET), STI, École Polytechnique Fédérale de Lausanne, 1015 Lausanne, Switzerland
[2]Department of Physics, University of Oxford, Oxford OX1 3PU, United Kingdom

November 6, 2025

[*]Correspondence email: alan.bowman@physics.ox.ac.uk
[†]Correspondence email: giulia.tagliabue@epfl.ch

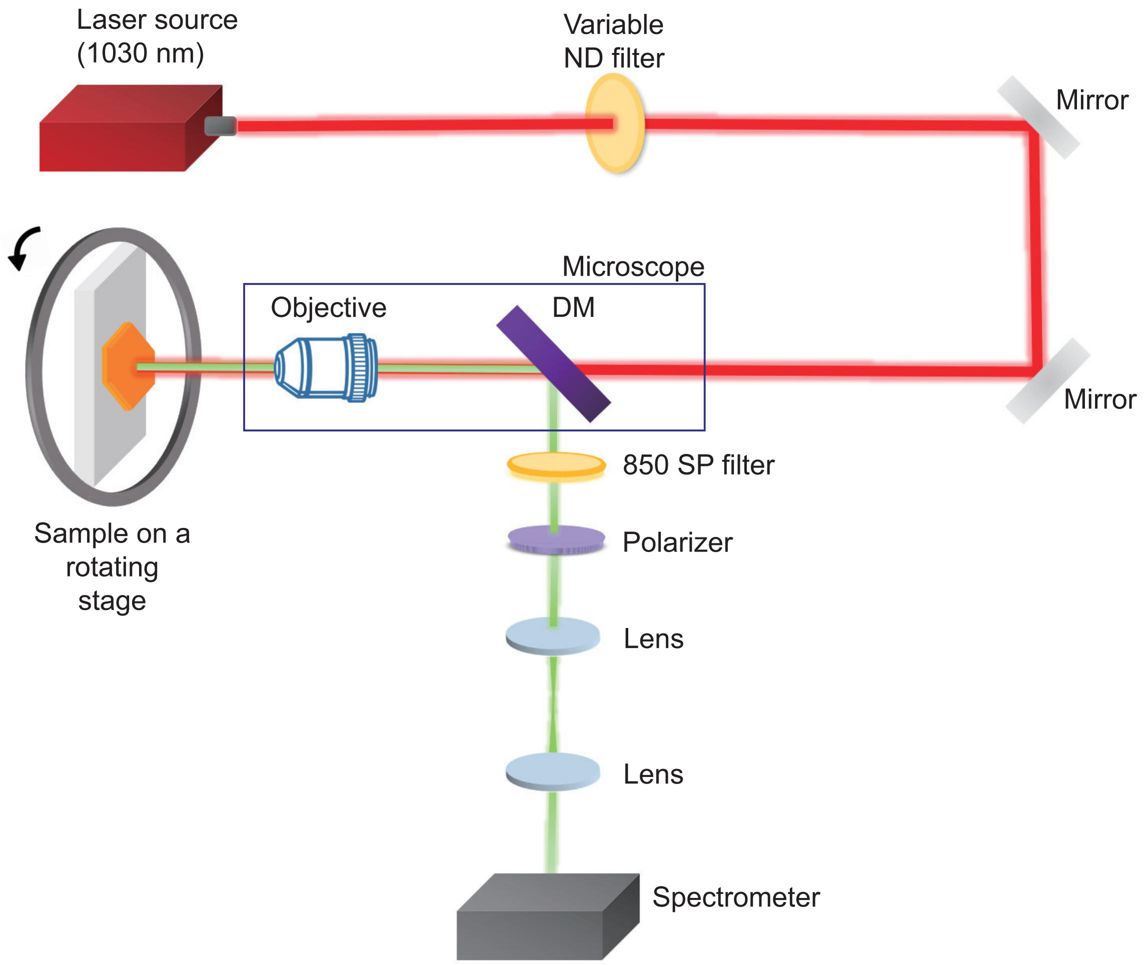

Figure S1: Schematic of the setup used for SHG measurements.The measurements were recorded using a microscope (Nikon Eclipse T2) coupled to a spectrometer (Princeton Instruments Spectra Pro HRS-500). A 1030nm femtosecond laser (NKT Origami) was used as excitation source and focused onto the sample by a 60x objective (Nikon S Plan Fluor ELWD, NA=0.7). An 750nm dichroic mirror (DM) was used to separate the fundamental and emitted signals. SP: Shortpass, ND: Neutral density. For the polarization-resolved measurements, a polarizer was inserted to the collection path.

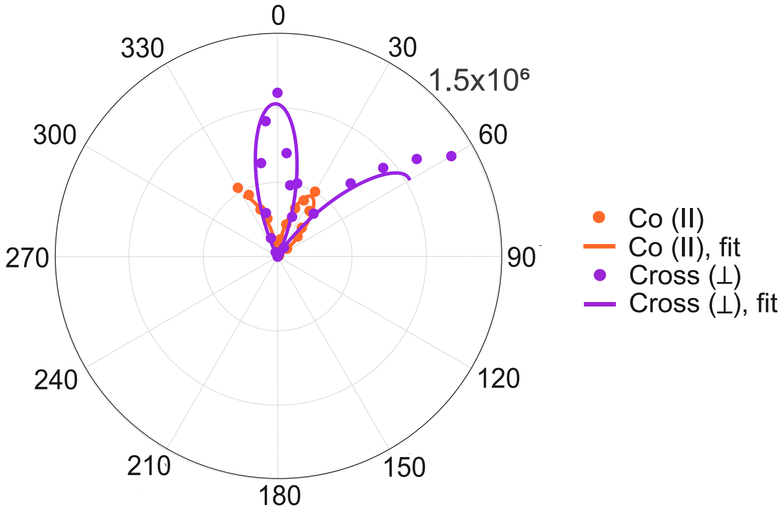

Figure S2: Polarization-resolved SHG measurements from a monocrystalline copper microflake in 0-90° range used for Fourier imaging measurements. Polar plot of SHG intensity as a function of detection polarization angle for co-polarized (orange) and cross-polarized (purple) emission, acquired at fixed incident polarization under 2 mW/μm^2 beam intensity.

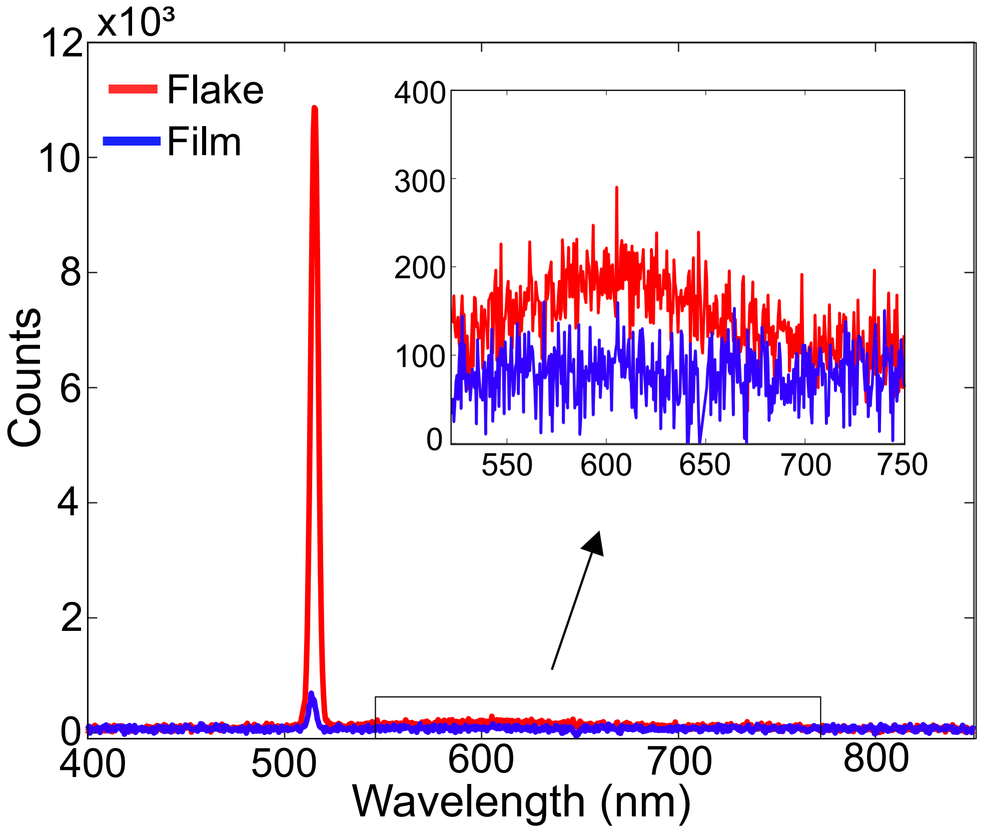

Figure S3: Relative SHG intensities for monocrystalline microflakes (red) and polycrystalline films (blue) when exciting at 1030 nm with 2.15 mW/μm^2 beam intensity. Inset shows expanded view of the two-photon photoluminescence response of the microflake in the 550-750 nm range. Small spot-to-spot and thickness dependent variation observed in the TPL strength wasn't thoroughly explored as it's not the main focus of this study.

a

b

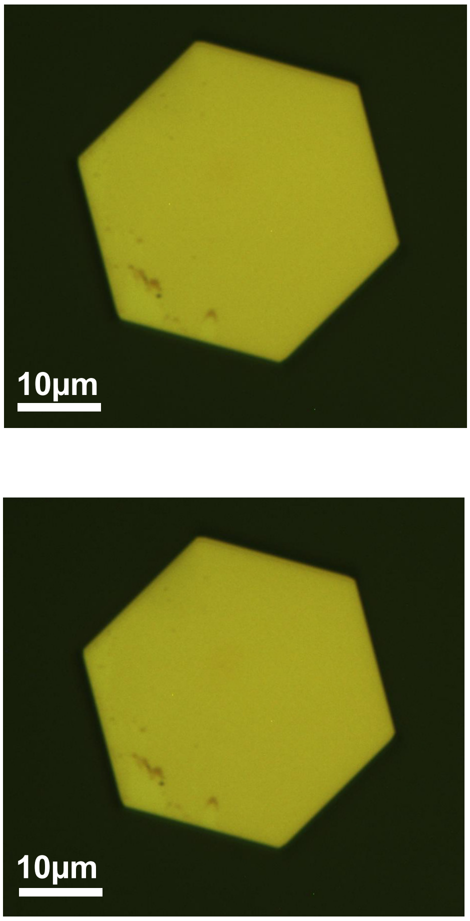

Figure S4: Bright-light images of the Cu microflake (a) before and (b) after 3 hours of continuous illumination under 2 mW/μm^2, showing no visible change to the flake surface under prolonged exposure.
